# A biphasic reduction in a measure of palatability following sucrose consumption in mice

**DOI:** 10.1016/j.physbeh.2017.11.019

**Published:** 2018-02-01

**Authors:** Jasmin A. Strickland, Joseph M. Austen, David J. Sanderson

**Affiliations:** Department of Psychology, Durham University, Science Site, South Road, Durham DH1 3LE, UK

**Keywords:** Feeding behavior, Licking, Memory, Adaptation, Hedonia, Satiety

## Abstract

Consumption of foods results in a transient reduction in hedonic value that influences the extent and nature of feeding behavior. The time course of this effect, however, is poorly specified. In an initial experiment, using an analysis of the microstructure of licking in mice we found that consumption of sucrose led to a rapid reduction in lick cluster size, a measure of palatability, which recovered after 10 min, but reemerged 60 min after initial consumption. We then replicated the finding that lick cluster size is reduced after 60 min, but not 10 min, under conditions in which a number of potential behavioural confounds were removed. In Experiment 2 the effect was replicated using a between-subjects design that ruled out the possibility that the effect was a specific consequence of the within-subjects procedures used in the first experiment, in which mice may have come to expect sucrose at different time points within the feeding session. While Experiments 1 and 2 confounded the time between periods of access to sucrose with time since the start of the feeding session, this confound was removed in Experiment 3, and, similar to the previous experiments, it was found that a second reduction in palatability occurred after 60 min. Therefore, the effect was dependent only on the time since the previous exposure to sucrose, demonstrating that sucrose consumption initiates a biphasic reduction in palatability. The reduction in lick cluster size after 60 min was not typically accompanied by a reduction in consumption suggesting that the more slowly developing reduction in the palatability measure was not simply a consequence of post-ingestive satiety. The cause of the biphasic change is not yet clear, and may reflect independent processes or the consequence of a single process that initiates multiple changes in palatability over time.

## Highlights

• Recent consumption of a palatable food results in a reduction in palatability.
• The time course of recovery of palatability after sucrose consumption was tested.
• Palatability was measured using the mean lick cluster size.
• Recent consumption of sucrose resulted in a biphasic change in lick cluster size.

## 1 Introduction

Identifying the factors involved in the short-term reduction of feeding is important for understanding the potential causes of overeating. Thus, it has been proposed that overeating, and ultimately obesity, may occur due to a failure to habituate to properties of foods during a meal [10], [15]. While the rate of consumption during continuous access to food (e.g., within a meal) decreases, the palatability of food also rapidly decreases [6], [13], [17]. This suggests that initial reductions in consumption may reflect reduced palatability, while subsequent reductions in consumption, that ultimately lead to the cessation of eating, may reflect post-ingestive satiety. The short-term reductions in palatability that occur as a consequence of recent consumption likely reflect sensory adaptation or habituation due to the influence of short-term memory [16]. Although the reductions in palatability that occur as a consequence of consumption of food rapidly recover, the time course of this recovery is unknown.

Examining rapid changes in palatability in humans is difficult due to the requirement for repeated self-reporting of hedonic responses. It is possible, however, to measure rapid changes in palatability in rodents by analysis of the microstructure of licking. Rodents drink by making by a series of licks in quick succession (a lick cluster) with each cluster separated by pauses typically > 0.5 s [8]. The number of licks in a cluster (lick cluster size) provides a measure of palatability that is dissociable from levels of consumption, as measured by the overall total number of licks. Thus, lick cluster sizes increase monotonically as a function of sucrose concentration, whereas the total number of licks follows an inverted U-shaped function with licking being maximal for intermediate sucrose concentrations and being lower for both high and low concentrations [5], [8], [18]. Therefore, while rodents will drink more of intermediate concentrations of sucrose than low concentrations, potentially due to the differences in palatability of the concentrations, they drink less of high concentrations than intermediate concentrations. Although this may indicate that they find high concentrations of sucrose less palatable than low concentrations, the reduction in consumption may also reflect the increased satiety caused by high concentrations. The fact that lick cluster sizes increase monotonically as a function of sucrose concentration indicates that the reduction in consumption of high sucrose concentrations does likely reflect increased satiety, and that lick cluster size provides a purer measure of palatability. Indeed, manipulations that affect lick cluster size typically also affect tests of taste reactivity (orofacial responses) in a similar manner, suggesting that these behavioural measures in rodents can be used to gauge the hedonic value of a substance (see [9], for a detailed discussion). Importantly, lick cluster sizes in mice are sensitive to a variety of memory manipulations [3], [5] suggesting that they provide a measure of experience-dependent changes in palatability.

We have previously investigated changes in lick cluster size in mice during a 10-min period of exposure to 16% sucrose and found that lick cluster sizes rapidly reduced during that period, with a significant reduction in the mean lick cluster size between the first and second minutes of exposure [19]. Furthermore, massing access to sucrose (e.g., one 5-min access) leads to lower lick cluster sizes than spacing of access (e.g., five 1-min periods of access each separated by four minutes) despite not affecting the overall levels of consumption [19]. Similar effects have been seen in brief access tests in mice in which consecutive 1-min periods of access to sucrose were separated by a 5-min interval [11]. These results suggest that palatability changes over exposure as a function of the time since the last period of consumption. The purpose of the present study was to examine the time course of the effect of recent consumption of sucrose on palatability and consumption. While it may be expected that recent consumption of sucrose will lead to a short-term reduction in palatability that recovers given a sufficient period of time, we instead found that, surprisingly, there was a biphasic reduction in palatability (Experiment 1).

In Experiment 1 mice received sessions in which they were allowed two 1-min periods of access to 16% sucrose. The interval between the exposures varied across sessions and could be 5 s, 10 min or 60 min. These intervals were chosen because our unpublished observations suggested that the reductions in lick cluster size are rapid, and, therefore, will be present after 5 s, but will have likely recovered after 10 min, and any effect should be minimal after 60 min. As expected, sucrose consumption led to a rapid decline in palatability when measured after 5 s, but there was no effect of recent consumption when the periods of feeding were separated by 10 min. Surprisingly, however, there was a reduction in palatability after a 60-min interval.

The finding that sucrose consumption led to a reduction in palatability after 5 s and 60 min, but not 10 min, was not anticipated. Therefore, the purpose of the subsequent experiments reported here was to test the reliability of the effect under conditions that rule out particular accounts. Experiment 2 replicated the effect of reduced palatability after 60 min but not 10 min using a between-subjects design that ruled out the possibility that reduced palatability after 60 min was a frustrative nonreward effect caused by extinction of the temporal expectation of sucrose. Experiment 3 replicated the finding of reduced palatability after 5 s and 60 min, but not 10 min, using a procedure that matched the time since the start of the feeding session, ruling out the possibility that the reduction in palatability after 60 min was due to the length of exposure to experimental test conditions.

## 2 Methods

### 2.1 Subjects

In Experiment 1, 12 experimentally naive C57BL/6 mice (six male, six female) bred in the Life Sciences Support Unit at Durham University were used. The mice were between five and seven months old at the start of testing and weighed between 15.8 g and 26.4 g. In Experiment 2, 24 experimentally naive female C57BL/6 mice from Charles River UK were used. They were approximately ten weeks old at the start of testing, weighing between 17.1 g and 20.8 g. In Experiment 3, 48 female C57BL/6 mice bred in the Life Sciences Support Unit at Durham University were used. The mice had previously received unrelated feeding procedures and were experienced at consuming sucrose solutions in the testing apparatus. They were between three and eight months old at the start of testing and weighed between 16.8 g and 26.3 g. Mice were caged in groups in a temperature controlled housing room with a 12 h light-dark cycle. Testing was conducted during the light period. During testing mice were motivated to consume the sucrose solution by being maintained at 85% of their free-feeding body weights. Using this method, we have previously shown that palatability, as measured by lick cluster size, can be manipulated by sucrose concentration, negative contrast, and habituation effects [3], [4], [5]. Mice had ad libitum access to water in their home cages. All procedures were in accordance with the United Kingdom Animals (Scientific Procedures) Act 1986 and were approved by the UK Home Office under project license number PPL 70/7785.

### 2.2 Apparatus

A set of eight identical operant chambers (interior dimensions: 21.6 × 17.8 × 12.7 cm; ENV-307 W, Med Associates, Inc., Fairfax, VT, USA), enclosed in sound-attenuating cubicles (ENV-022V, Med Associates) were used. The operant chambers were controlled by Med-PC IV software (Med Associates). The side walls were made from aluminium, and the front and back walls and the ceiling were made from clear Perspex. The chamber floors each comprised a grid of 24 stainless steel rods (0.32 cm diameter), spaced 0.79 cm apart and running perpendicular to the front of the chamber (ENV-307W-GFW, Med Associates). Retractable sippers (ENV-352AW, Med Associates) and a small hole in one wall of each chamber allowed graduated pipettes to be extended into, and retracted from, the chambers. The graduated pipettes (10:0.1 ml) allowed measurement of consumption by comparing the volumes before and after testing. Contact lickometer controllers (ENV-250, Med Associates) allowed contacts between the mice and the graduated pipettes to be recorded at a resolution of 0.01 s. A fan (ENV-025F, Med Associates) was located within each of the sound-attenuating cubicles and was turned on during sessions. Sucrose solutions were made weight/volume with commercially available sucrose in distilled water.

### 2.3 Procedure

#### 2.3.1 Experiment 1

Mice received two 1-min periods of access to 16% sucrose solution (0.47 mol/l) per session. This was achieved by inserting the sipper tube into the chamber and then withdrawing it at the end of each 1-min period. The first period occurred 5 min after the start of the session. After the first period of access the second occurred after one of three possible intervals: 5 s, 10 min or 60 min. The session ended immediately after the second period of access to sucrose. Mice received fifteen sessions, one per day, with five of each interval (5 s, 10 min, and 60 min). The order of intervals across sessions was randomised with the constraint that on any given session one third of the mice received each interval and over each block of three sessions each animal received one session with each interval.

#### 2.3.2 Experiment 2

Mice received six sessions in which they were allowed two 1-min periods of access to 16% sucrose (0.47 mol/l). For one group of mice (N = 12) the two periods were separated by a 10-min interval, and for another group (N = 12) the interval was 60 min. All other details were the same as Experiment 1.

#### 2.3.3 Experiment 3

**Fig. 1:**
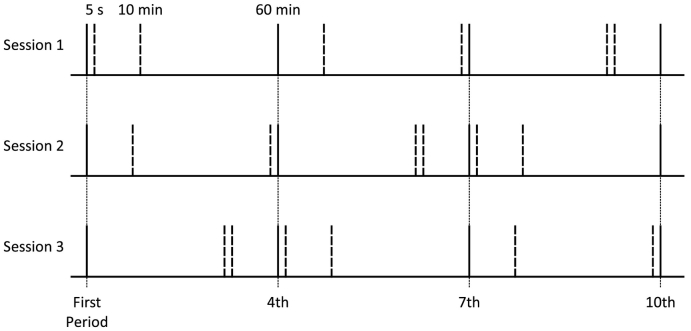
Example timelines of the order of periods of access to sucrose. Each period of access is represented as a vertical line. After the first period of access, subsequent periods were separated by 5 s, 10 min and 60 min with one of each interval every three periods of access. This resulted in the first, fourth, seventh and tenth periods of access occurring at the same time point within the session regardless of the order of the inter-period intervals. These periods that occurred at time-matched points within the session are represented as solid vertical lines, whereas periods of access that weren't time-matched are represented by dashed vertical lines. Mice received three sessions and across sessions each interval (5 s, 10 min and 60 min) was assessed once on the fourth, seventh and tenth period.

Mice received three sessions in which they were allowed ten 1-min periods of access to 16% sucrose (0.47 mol/l). After the first period of access the subsequent periods occurred after intervals of 5 s, 10 min or 60 min, with three of each interval per session and one of each interval every block of three periods of access after the initial period of access (see Fig. 1 for examples of the timelines of events within a session). This procedure ensured that the fourth, seventh and tenth periods of access occurred 72 min and 5 s, 145 min and 10 s, and 218 min and 15 s after the first period of access respectively, regardless of the order of the intervals used between each period. For each session one of each interval immediately preceded the fourth, seventh and tenth periods of access. Across the three sessions, each interval (5 s, 10 min and 60 min) preceded each of the fourth, seventh and tenth periods of access once. This procedure ensured that across sessions the effect of each interval was assessed after the same amount of time had elapsed since the start of the session. Mice received the order of intervals in one of six combinations (N = 8 per interval order) that ensured that the interval used for the fourth, seventh and tenth period of access was equally often 5 s, 10 min and 60 min across mice for each of the individual sessions, and that the order of intervals was balanced across mice. All other details were the same as Experiment 1.

### 2.4 Data and statistical analyses

For all experiments the total number of licks and mean number of licks per cluster (lick cluster size) were measured. A lick cluster was defined as a series of two or more licks made with < 0.5 s between the end of one lick and the start of the next. Therefore, when there was a pause in licking of at least 0.5 s the lick cluster was considered to have ended. We have used this lick cluster criterion in a number of previous studies in mice [3], [4], [5]. Importantly, when we have examined the effect of sucrose concentration on lick cluster size there is very little difference between the 0.5 s lick cluster criterion and the use of a 0.25 s or 1 s criterion, because the vast majority of licks that are separated by at least 0.25 s are actually separated by gaps of at least 1 s [5]. Furthermore, within a lick cluster the interval between the end of one lick and the start of the next is typically < 0.1 s [4], therefore, the 0.5 s lick cluster criterion used in the present study far exceeds this duration making it unlikely that it underestimates lick cluster sizes.

For each mouse, mean lick cluster sizes following particular intervals were calculated by dividing the mean number of licks that were made within lick clusters in the period immediately following that interval across sessions by the mean number of lick clusters completed within that same period across sessions. This method approximates the mean lick cluster size for a particular time period, but potentially leads to a mean that differs to an extent from the mean of the lick clusters that were started and completed within the time period. For Experiment 3 analysis of licking behavior was restricted to performance on the fourth, seventh and tenth periods of access to sucrose. This ensured that, across sessions, licking after the different intervals (5 s, 10 min and 60 min) was compared at equivalent time points since the start of the session.

All data were analysed using one-way or multifactorial ANOVA. Interactions were analysed with simple main effects analysis using the pooled error term from the original ANOVA, or separate repeated measures ANOVA for within-subjects factors with more than two levels. Where sphericity of within-subjects variables could not be assumed, the Greenhouse-Geisser correction was applied. Post-hoc comparisons were made using the Bonferroni correction to control for the family-wise error rate.

## 3 Results

### 3.1 Experiment 1

**Fig. 2:**
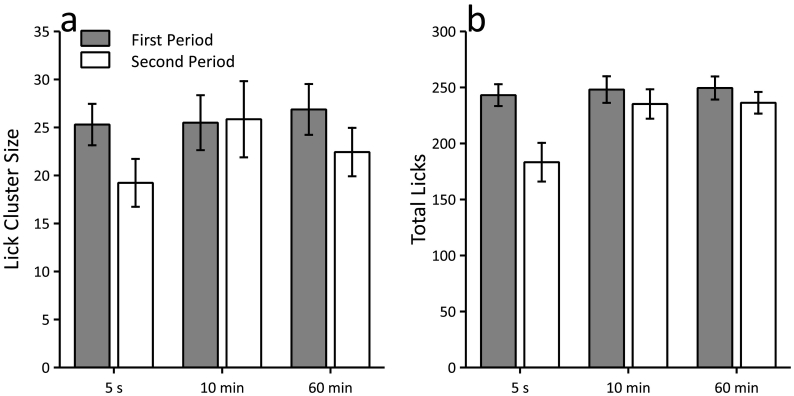
Sucrose consumption results in reduced lick cluster size after 5 s and 60 min, but not 10 min, in a procedure in which the test interval was manipulated within-subjects. A reduction in the number of licks was evident after each interval. Panels a and b show the mean lick cluster size and the total number of licks, respectively, for the first and second 1-min periods of access to 16% sucrose, which were separated by 5 s, 10 min or 60 min. Error bars indicate ± SEM.

When the periods of access to sucrose were separated by 5 s or 60 min mice showed a reduction in lick cluster size during the second period compared to the first (see Fig. 2a). This reduction did not occur when the interval was 10 min. This pattern of results was confirmed by a significant period (first, second) by interval (5 s, 10 min, 60 min) interaction (F(2,22) = 5.71, p = 0.010). Simple main effects analysis revealed that the effect of period was significant when there was a 5-s interval (F(1,11) = 12.79, p = 0.004) and 60-min interval (F(1,11) = 14.73, p = 0.003), but not 10-min interval (F < 1, p > 0.80). There was a significant effect of interval for the second period (F(2,22) = 4.94, p = 0.017). None of the pairwise comparisons, however, reached significance (smallest p = 0.067, Bonferroni corrected). The effect of interval was not significant for the first period (F < 1, p > 0.50).

Simple main effects analysis of the significant interaction demonstrated that there was a reduction in lick cluster size after 60 min, but not after 10 min. This pattern of results was surprising given that it was assumed that the effect of recent sucrose consumption on lick cluster size should reduce over time. In order to test directly whether the reduction in lick cluster size after 60 min was greater than after 10 min, the lick cluster sizes during the second period were subtracted from the first period for both intervals (10 and 60 min) so that the size of the difference between the two periods could be compared between intervals. It was found that the reduction in lick cluster size after 60 min was greater than after 10 min (60 min, mean = − 4.44 ± 1.16 SEM; 10 min, mean = 0.36 ± 1.72 SEM; t(11) = 2.45, p = 0.032).

There was a significant period by interval interaction for the total number of licks (F(2,22) = 16.28, p < 0.001, see Fig. 2b). The simple main effects analysis revealed that there was a significant reduction in licks in the second period compared to the first for all intervals (smallest F(1,11) = 5.08, p = 0.046). There was a significant effect of interval for the second period (F(2,22) = 16.27, p < 0.001) with mice making fewer licks in the 5 s condition compared to both the 10-min and 60-min conditions (largest p = 0.005, Bonferroni corrected). There was no difference between the 10-min and 60-min conditions (p > 0.90). There was no significant effect of interval for the first period (F < 1, p > 0.70).

### 3.2 Experiment 2

The purpose of Experiment 2 was to rule out the possibility that the reduction in palatability after 60 min in Experiment 1 was a specific consequence of the within-subjects procedure in which mice were exposed to different schedules of access to sucrose across sessions. Mice were allowed two 1-min periods of access to sucrose per session, but for half of the mice the interval between the periods of access was always 10 min, and for the other half the interval was always 60 min.

**Fig. 3:**
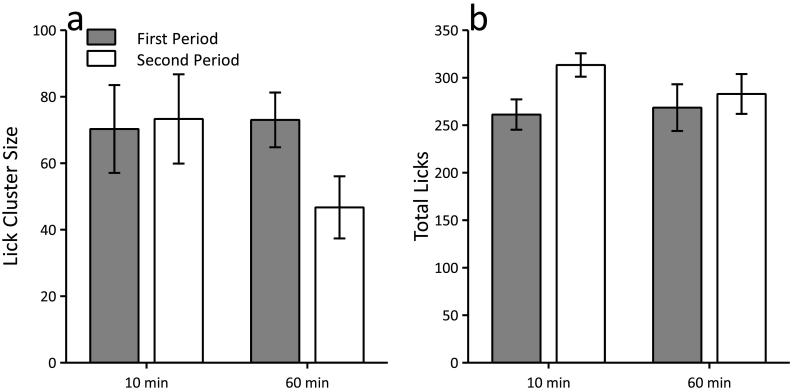
Sucrose consumption results in reduced lick cluster size after 60 min, but not 10 min, in a procedure in which the test interval was manipulated between-subjects. The number of licks increased after 10 min but not 60 min. Panels a and b show the mean lick cluster size and the total number of licks, respectively, for the first and second 1-min periods of access to 16% sucrose, which were separated by 10 min or 60 min. Error bars indicate ± SEM.

Mice that received the two periods of access to sucrose separated by 10 min failed to show a reduction in lick cluster size in the second period compared to the first, but for the mice in which the periods were separated by 60 min there was an attenuation in lick cluster size over periods (see Fig. 3a). This pattern of results was confirmed by a significant period (first, second) by interval (10 min, 60 min) interaction (F(1,22) = 6.68, p = 0.017). Simple main effects analysis confirmed that there was a significant effect of period in the 60-min condition (F(1,22) = 12.30, p = 0.002), but not for the 10-min condition (F < 1, p > 0.80). In addition, there was no significant effect of interval for the first period (F < 1, p > 0.80), but there was for the second period (F(1,22) = 4.49, p = 0.046). There was also a significant period by interval interaction for the total number of licks (F(1,22) = 6.94, p = 0.015, see Fig. 3b). This was due to mice in the 10-min condition making a greater number of licks in the second period compared to the first (F(1,22) = 22.90, p < 0.001). This was not the case for the 60-min condition (F(1,22) = 1.11, p = 0.30). There was no difference between intervals for either period (largest F(1,22) = 2.05, p = 0.17).

### 3.3 Experiment 3

**Fig. 4:**
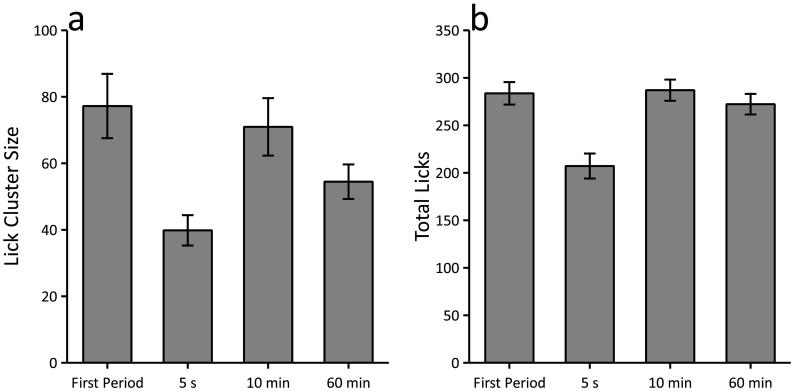
Sucrose consumption results in reduced lick cluster size after 5 s and 60 min, but not 10 min, in a procedure that matched the cumulative exposure to the feeding context at the time of consumption. The number of licks was reduced after 5 s compared to 10 and 60 min, but there was no difference between 10 and 60 min. Panels a and b show the mean lick cluster size and the total number of licks, respectively, for the first period and for the 1-min periods of access to 16% sucrose that followed an interval of 5 s, 10 min or 60 min. Error bars indicate ± SEM.

Comparing the 4th, 7th and 10th periods, which were matched for the time elapsed since the start of the session, mice made smaller lick clusters if the period was preceded by an interval that was 5 s or 60 min, but not if the preceding interval was 10 min (Fig. 4a). These results were confirmed by a significant effect of interval (F(2,94) = 14.61, p < 0.001). Lick clusters were significantly smaller in the 5-s and 60-min conditions compared to the 10-min condition (largest p = 0.025, Bonferroni corrected), and lick clusters were smaller in the 5-s condition compared to the 60-min condition (p = 0.008, Bonferroni corrected). There was also a significant effect of interval on the total number of licks (F(2,94) = 33.33, p < 0.001, see Fig. 4b). Mice made fewer licks in the 5-s condition compared to both the 10-min and 60-min conditions (p-values < 0.001, Bonferroni corrected). There was no significant difference between the 10-min and 60-min conditions (p = 0.072, Bonferroni corrected).

In the previous analyses, we tested the effect of interval (5 s, 10 min and 60 min) by collapsing across the period of access within the session (4th, 7th and 10th) across sessions. There was a main effect of interval on lick cluster size showing that lick cluster sizes were lower after 5 s and 60 min compared to 10 min. It is possible, however, that this effect was driven by performance early on in the session when relatively little consumption had occurred and may not be present later on when post-ingestive feedback may affect performance. In order to test this possibility, we repeated the analyses and included period (4th, 7th and 10th) as a factor. Fifteen mice failed to complete a lick cluster for each interval (5 s, 10 min and 60) at each period (4th, 7th and 10th). Therefore, the analysis was carried on the remaining 33 mice. As for the previous analyses we found an effect of interval (F(2,64) = 11.35, p < 0.001) and post-hoc analyses confirmed that lick cluster sizes were significantly lower for the 5 s and 60 min intervals compared to the 10 min interval (largest p = 0.045, Bonferroni corrected) and lick clusters were significantly lower for the 5 s interval compared to the 60 min interval (p = 0.029, Bonferroni corrected). There was, however, no significant effect of period (F(2,64) = 1.61, p = 0.21) and no interaction with interval (F(4,128) = 1.43, p = 0.23).

A similar analysis was carried out for the total licks measure, but now all 48 mice were included. The total number of licks did not significantly change over periods (F(2,94) = 2.90, p = 0.060), but there was an interaction between interval and period (F(4,188) = 2.62, p = 0.049). This interaction reflected the fact that the effect of interval on total licks was apparent in the 4th and 10th periods (largest p = 0.001), but not for the 7th (p = 0.11). Post-hoc analyses confirmed that in the 4th and 10th periods the total number of licks was lower for 5 s interval than both the 10 min and 60 min intervals (largest p = 0.015, Bonferroni corrected) and the total number of licks did not differ between the 10 min and 60 min intervals (smallest p = 0.29, Bonferroni corrected).

## 4 Discussion

The results demonstrate that recent consumption of sucrose in mice leads to separate reductions in palatability that develop over different time courses. The first reduction occurred rapidly and was evident when there was a 5-s interval between successive 1-min periods of access to sucrose. This attenuation of palatability was transient, however, and was not evident with a 10-min interval. The second reduction in palatability developed more slowly and was evident with a 60-min interval.

There was large variation in lick cluster size across experiments. Specifically, the lick cluster sizes in Experiment 1 were much smaller than in Experiments 2 and 3. Other than the procedural differences between experiments a number of other factors may have led to this variation (e.g., age, breeding location, previous experience of mice etc.). Despite the differences in the baseline size of lick clusters across experiments, all three experiments showed that the reduction in lick cluster size after 60 min was greater than after 10 min.

The reduction in palatability that was evident 60 min after initial consumption of sucrose may have been due to an emotional response caused by the surprising omission of sucrose during the 60-min interval. Thus, in Experiment 1, as a consequence of exposure to the different schedules of sucrose access, mice may have learnt to time the presentations of sucrose across the duration of the session. Given that mice were exposed to the different intervals across sessions, this would have led to mice expecting sucrose 5 s, 10 min or 60 min after the initial period of access. Consequently, in sessions when a 60-min interval was used, the surprising omission of sucrose after 5 s and 10 min may have led to a frustrative nonreward effect in which the omission of reward induced an aversive state [1], [2], which may have reduced the perceived palatability of sucrose when subsequently presented. The results of Experiment 2 rule out this account. The possibility of a frustrative nonreward effect was avoided by subjecting mice to only one interval between exposures to sucrose (either 10 min or 60 min) so that they received a schedule of access to sucrose that was consistent over sessions. It was found, however, that there was still a reduction in palatability after a 60-min interval but not a 10-min interval. Therefore, the results of Experiment 2 demonstrate that the results of Experiment 1 were not a specific consequence of the within-subjects procedure that was used.

The reduction in palatability after 60 min but not after 10 min may have been due to differences in the length of exposure to the experimental context. Thus, it is possible that 60 min of exposure to the context (the chamber in which sucrose was presented) induced a negative state through, for example, stress [12], potentially caused by social isolation [14], and this may have been the cause of reduced palatability rather than prior consumption of sucrose. In Experiment 3, in order to rule out the duration of exposure to the experimental context as a confounding factor, mice received exposures to sucrose that were separated by 5 s, 10 min or 60 min, but the effect of the different intervals was assessed at the same time points within the session. It was still found that mice showed a reduction in palatability if sucrose had been consumed 5 s and 60 min before, but not if prior consumption had happened 10 min before.

While the reduction in palatability at 5 s may reflect sensory adaptation, or simply motor fatigue, these processes cannot explain the reduction in palatability after 60 min. Such effects are short-term, and, indeed, there was no reduction in palatability after 10 min suggesting that any effect of sensory adaptation or fatigue had recovered by this point. An alternative account of the reduction in palatability after 60 min is that there was sufficient time for post-ingestive feedback to result in satiety. Indeed, it would be informative to know whether the reduction in lick cluster size after 60 min would still occur if post-ingestive satiety was reduced by using a weaker concentration of sucrose or a non-caloric sweetener. There may be reasons, however, to doubt post-ingestive satiety as a likely explanation. Although there was a reduction in consumption (as indicated by the number of licks) after 60 min in Experiment 1, this effect was not replicated in Experiments 2 and 3, suggesting that the reduction in palatability can occur in the absence of changes in overall consumption. There is, however, the potential that a reduction in consumption was masked by a cue-potentiated feeding effect caused by an association between the context and sucrose. Experiment 3 demonstrated that the pattern of reduced lick cluster size after 60 min, but not 10 min, was not affected by the time at which it occurred during a prolonged session of consumption, suggesting that the effect was independent of any possible post-ingestive influences on consumption. Therefore, any potential reduction in consumption after 60 min (e.g., in Experiment 1) may be a consequence of a reduction in palatability, rather than the reverse being true. This may suggest that the decline in palatability after 60 min reflects a form of sensory-specific satiety [16] that primarily reflects changes in palatability or “liking” rather than “wanting” (i.e., motivation to consume sucrose) [7].

The biphasic change in palatability that occurs as a consequence of sucrose consumption may reflect multiple processes that affect palatability at different rates. For example, it has been suggested that stimuli activate separate representations of their specific sensory properties and their affective properties and that the activation of these separate memories affects behavior over different time courses [20]. It is, however, possible that the biphasic change reflects a single process that initiates a succession of changes such that palatability fluctuates over time. Currently it is not possible to choose between these two potential accounts.

## 5 Conclusion

In conclusion, we have found that lick cluster size, a measure of palatability, follows a biphasic reduction after brief consumption of sucrose. The experiments reported here demonstrate that the effect is robust and is not specific to a number of behavioural variables. It is not yet clear, however, whether the biphasic response is specific to sucrose, the concentration of sucrose or the particular time points used to measure the changes in lick cluster size over time. Future work needs to address the generalizability of the effect as well as the cause of the more slowly developing change in palatability after 60 min.

## Funding

The work was funded by grants from the Royal Society (RG130005) and BBSRC (BB/M009440/1) awarded to DJS.
